# KPG Index versus OPG Measurements: A Comparison between 3D and 2D Methods in Predicting Treatment Duration and Difficulty Level for Patients with Impacted Maxillary Canines

**DOI:** 10.1155/2014/537620

**Published:** 2014-07-09

**Authors:** Domenico Dalessandri, Marco Migliorati, Luca Visconti, Luca Contardo, Chung How Kau, Conchita Martin

**Affiliations:** ^1^Department of Orthodontics, School of Dentistry, University of Brescia, Piazzale Spedali Civili 1, 25123 Brescia, Italy; ^2^Department of Medical, Surgical and Health Sciences, School of Dentistry, University of Trieste, Piazza Ospitale 1, 34129 Trieste, Italy; ^3^Department of Orthodontics, School of Dentistry, University of Genova, Viale Benedetto XV 6, 16132 Genova, Italy; ^4^Department of Orthodontics, School of Dentistry, University of Alabama, Birmingham, AL 35233, USA; ^5^Department of Stomatology IV, School of Dentistry, Complutense University of Madrid, Plaza Ramòn Cajal s/n, 28040 Madrid, Spain

## Abstract

*Aim*. The aim of this study was to test the agreement between orthopantomography (OPG) based 2D measurements and the KPG index, a new index based on 3D Cone Beam Computed Tomography (CBCT) images, in predicting orthodontic treatment duration and difficulty level of impacted maxillary canines. *Materials and Methods*. OPG and CBCT images of 105 impacted canines were independently scored by three orthodontists at *t*_0_ and after 1 month (*t*_1_), using the KPG index and the following 2D methods: distance from cusp tip and occlusal plane, cusp tip position in relation to the lateral incisor, and canine inclination. Pearson's coefficients were used to evaluate the degree of agreement and the *χ* ^2^ with Yates correction test was used to assess the independence between them. *Results*. Inter- and intrarater reliability were higher with KPG compared to 2D methods. Pearson's coefficients showed a statistically significant association between all the indexes, while the *χ* ^2^ with Yates correction test resulted in a statistically significant rejection of independency only for one 2D index. *Conclusions*. 2D indexes for predicting impacted maxillary canines treatment duration and difficulty sometimes are discordant; a 3D index like the KPG index could be useful in solving these conflicts.

## 1. Introduction

Maxillary canines are the second most frequently impacted teeth after the third molars. Considering the not negligible prevalence of impacted canines, ranging from 0.9% up to 5% [1–3] and the difficulties sometimes related to their orthodontic treatment, several authors have been trying to elaborate prognostic indexes in order to foresee, during the diagnostic process, some important factors such as treatment rough duration and difficulty level [4, 5]. These indexes were all based on two-dimensional (2D) radiographs, such as OPG, occlusal, periapical, and lateral cephalograms, which are all characterized by the reduction of the examined volume into flat images, with a variable distortion of real dimensions and with different possible patient positioning errors, further affecting image quality and trustworthiness [6–8].

Recently, also thanks to the rapidly increasing availability of CBCT scanners and their present status of gold standard in three-dimensional (3D) dental and maxillofacial radiology [9–11] for both pathological [12–14] and healthy patients [15, 16], a 3D index was proposed classifying impacted maxillary canines treatment difficulty into four categories: easy, moderate, difficult, and very difficult [17]. The use of this index was found to be reliable, considering its high inter- and intrarater reliability [18], and with a good level of agreement with the orthodontist's perception of treatment difficulty [19]. Furthermore the accuracy of CBCT measurements [20, 21] and the possibility to reorientate with a visualization software the acquired volumes when patient malpositioning eventually occurred during images acquisition [22, 23] contribute to strengthen the reliability of KPG index. Anyway, as far as we know, no comparison was realized until now between classical well known 2D index and this new 3D index outcomes.

Thus, the aim of this study was to compare inter- and intrarater reliability of 2D versus KPG indexes and to evaluate their level of agreement in impacted maxillary canines rating.

## 2. Materials and Methods

OPG and CBCT exams of 90 subjects, 15 with bilateral impactions and 75 with unilateral impactions, coming from three different radiological centers (A, B, C), were randomly extracted from our database obtaining a sample of 105 impacted canines. These records were independently scored with both 2D and 3D indexes, after a calibration meeting, by three orthodontists at *t*_0_ and after 1 month (*t*_1_). After that, a joint measuring session was organized (*t*_2_) and these results were utilized for qualitative analysis: all discrepancies were resolved finding a common agreement.

30 patients (22 with unilateral and 8 with bilateral impacted maxillary canines) came from the radiological center A, where OPG images were obtained with an Orthophos XGplus Sirona digital machine set at 72 kV, 8 mA, and 15 seconds of exposure, while CBCT exams were realized with a NewTom 5G scanner set at 0.3 mm voxel and 15 × 15 cm* Field of View* (FOV) sizes, with a slice interval of 1 mm; 30 patients (27 with unilateral and 3 with bilateral impacted maxillary canines) came from the radiological center B, where OPG images were obtained with a Kodak 8000C digital machine set at 73 kVp, 12 mA, and 13.9 seconds of exposure, while CBCT exams were realized with a Kodak 9500 scanner set at 0.3 mm voxel and 15 × 9 cm FOV sizes, with a slice interval of 1 mm; and 30 patients (26 with unilateral and 4 with bilateral impacted maxillary canines) came from the radiological center C, where OPG images were obtained with an Instrumentarium OP100 digital machine set at 73 kV, 12 mA, and 17.6 seconds of exposure, while CBCT exams were realized with a Planmeca Promax Mid scanner set at 0.2 mm voxel and 16 × 9 cm FOV sizes, with a slice interval of 1 mm.

CBCT images, after Digital Imaging and Communications in Medicine (DICOM) files export, were visualized with the following software: NNT Viewer for radiological center A; Kodak Dental Imaging 3D-module software for center B; and Planmeca Romexis software for center C. OPG images were extracted from the original software, saved as JPEG files, and viewed using Windows Photo Viewer (Microsoft Corporation, Redmond, WA, USA). All the radiological images were visualized on a 16 : 9 27′′ Light Emitting Diodes (LED) backlighting monitor display (iMac, Apple, Cupertino, CA, USA) with a 2560 × 1440 pixel screen resolution.

### 2.1. KPG Index

**Figure 1:**
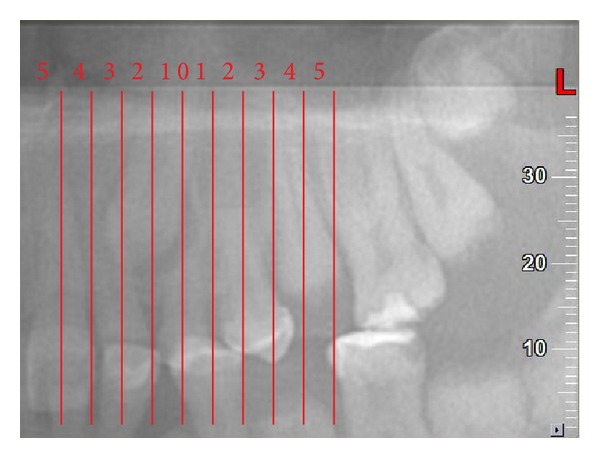
Mesiodistal position (*x*) for both cusp and root tips; panorex view. In this example *C*_*x*_ = 2 and *R*_*x*_ = 1.

**Figure 2:**
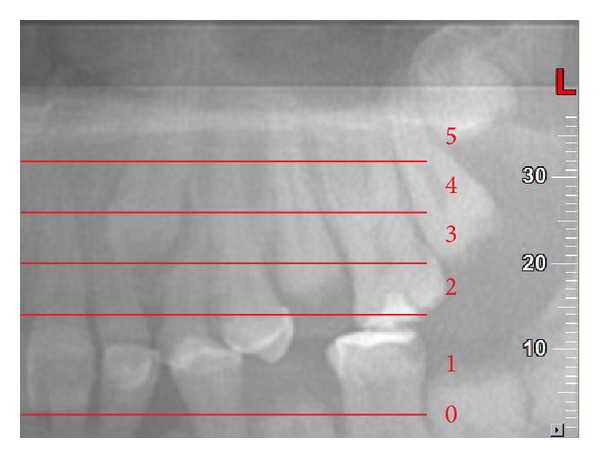
Vertical position (*y*) for cusp tip; panorex view. In this example *C*_*y*_ = 3.

**Figure 3:**
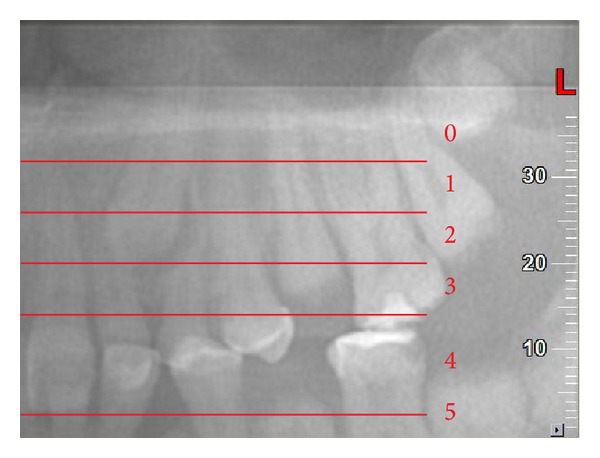
Vertical position (*y*) for root tip; panorex view. In this example *R*_*y*_ = 0.

**Figure 4:**
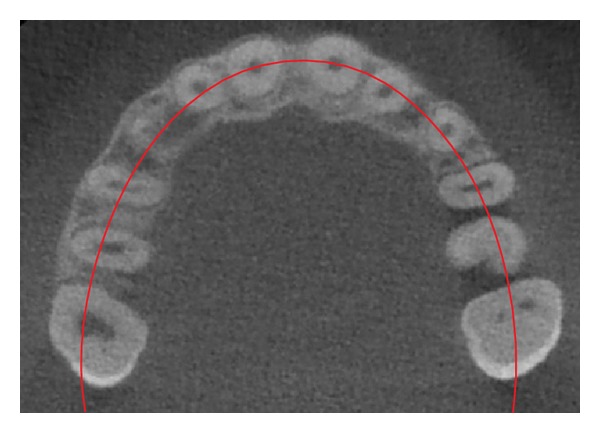
Occlusal reference arch (*z*); axial view. In this example *C*_*z*_ = 2 and *R*_*z*_ = 3, therefore the KPG index value is 11—moderate difficulty (2 + 1 + 3 + 0 + 2 + 3 = 11), in this study considered as shorter and easier.

KPG index was calculated adding together the scores, from 0 to 5, assigned to cusp tip and root tip on *x*, *y*, and *z* planes (Figures 1, 2, 3, and 4): in the original version scores in the range 0–9 fell into the category of easy, 10–14 were moderate, 15–19 were difficult, and 20–30 were extremely difficult; in the modified version the category of easy was reduced to 0–6 scores, extending the category of moderate from 7 to 14. In order to compare the KPG index with 2D indexes, these four categories were reduced to two, creating an easy-moderate category in the range 0–14 and a difficult-very difficult category in the range 15–30.

### 2.2.  2D Methods

**Figure 5:**
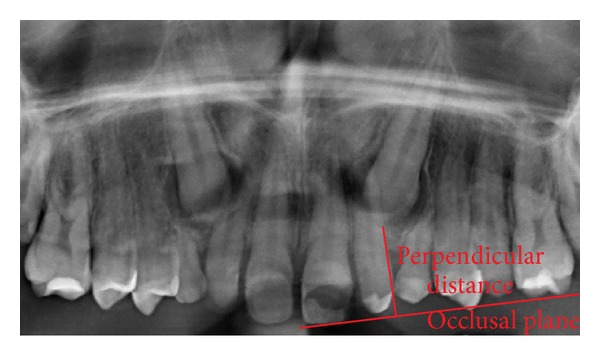
Vertical distance from the cusp tip perpendicularly to the occlusal plane, traced from the first upper molar to the central upper incisor. In this example, 14.8 mm, corresponding to a longer treatment according to Stewart.

**Figure 6:**
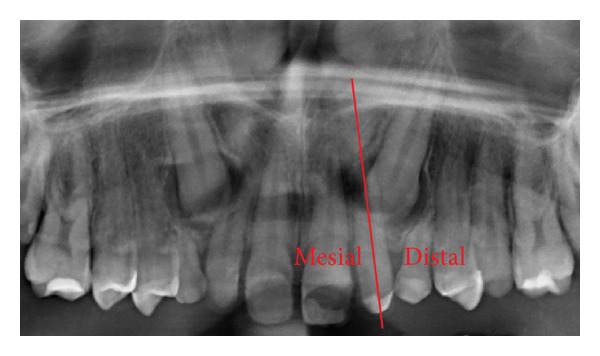
Mesiodistal position of the canine tip with respect to the adjacent teeth. In this example the canine is distal to the lateral, corresponding to an easier treatment according to Ericson and Kurol.

**Figure 7:**
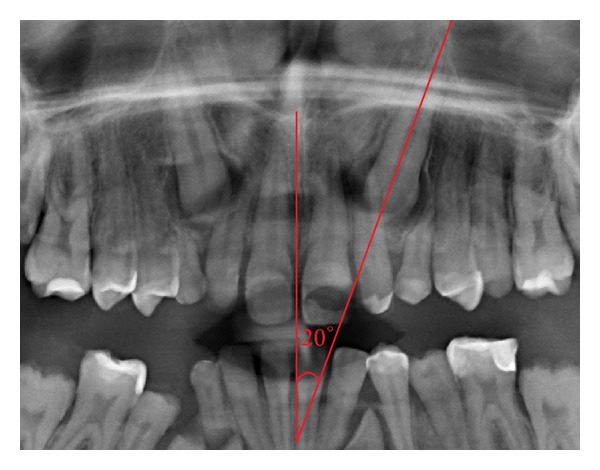
Canine inclination, *α*-angle, to a vertical line traced between the two central incisors. In this example *α*-angle is 20°.

After a literature review, we identified three different 2D measurements on OPG that were commonly used to predict treatment duration or difficulty degree when planning an impacted maxillary canine orthodontic treatment: the vertical distance from the cusp tip perpendicularly to the occlusal plane, traced from the first upper molar to the central upper incisor (Figure 5); the mesiodistal position of the canine tip with respect to the adjacent teeth (Figure 6); the canine inclination, *α*-angle, to a vertical line traced between the two central incisors (Figure 7).

According to Stewart et al. [4], vertical distances from the cusp tip perpendicularly to the occlusal plane measuring less than 14 mm were associated with shorter treatment duration, and that one measuring 14 mm or more was associated with longer treatment duration. Therefore, comparing this measurement with KPG index, we considered two categories: shorter treatment under 14 mm and longer treatment for 14 mm or more.

According to Ericson and Kurol [5], canines with cusp tip position in sectors 1-2, distal to the lateral incisor vertical midline, were considered easier to treat, compared to canines with a more mesial position, corresponding to sectors 3–5. Therefore, comparing this measurement with KPG index, we considered two categories: easier treatment when cusp tip was distal to the lateral incisor midline and difficult treatment when cusp tip was more mesially positioned.

According to Crescini et al. [24], every 5° of opening of the *α*-angle required approximately 1 more week of active orthodontic traction. It was not possible to identify a cut-off value between shorter and longer treatments; then this measurement was not compared with the KPG index.

### 2.3. Sample Description

The present study was based on filed CBCT exams (of both treated and untreated cases) randomly extracted from our database; that is, the exams were not expressly performed for our study aims but were prescribed based on clinical evaluations, pondered case by case, because of ectopic position of the canine. The CBCT examination was considered supplemental to conventional radiographic examination. Informed consent to undergo the additional radiographic examination and to use the material for future studies was obtained from all patients and parents/tutors.

### 2.4. Statistical Analysis

Inter- and intrarater reliability for both 2D and 3D methods were calculated, utilizing Cohen's kappa and Kendall's* W* coefficients, respectively. Both coefficients range from 0 to 1, with higher values indicating a stronger relationship: values ≤ 0.01 indicate poor agreement and values between 0.01 and 0.20 slight agreement, between 0.21 and 0.40 fair agreement, between 0.41 and 0.60 moderate agreement, between 0.61 and 0.80 substantial agreement, between 0.81 and 0.99 almost perfect agreement, and 1 perfect agreement.

The qualitative mean results (short or long, easy or difficult), obtained at *t*_2_ from these methods, were plotted using contingency tables, and Pearson's coefficients were calculated in order to evaluate the degree of agreement. Conversely, the *χ* ^2^ with Yates correction (or continuity correction) test was used to assess the independence between them.

The Pearson coefficient ranges from −1.0 to +1.0: −1.0 is a strong inverse relationship, 0 indicates no relationship, and +1.0 is a strong direct relationship. Values between 0.3 and 0.5 indicate a medium correlation, and between 0.5 and 1.0 a high correlation. We set statistical significance at 0.05 and we did not rely upon Pearson coefficient values when *P* > 0.05.

The *χ* ^2^ test compares the observed frequency with the expected frequency in each category in a contingency table. Even if our sample dimension was rather large, nevertheless, we decided to use a continuity correction such as the Yates correction, considering that we were approximating a continuous *χ* ^2^ distribution by discrete observations and that the 2 × 2 tables that we utilized only have one degree of freedom. Statistical significance was set at 0.05.

In our study, we set a null kappa value of 0.40; the level at which the kappa is statistically significantly different than the null value was set at 0.70 (a 0.30 difference should be the smallest difference tested); 80% power was selected and the expected proportion of positive ratings, based on our previous studies, was determined at 70%. The sample size for the 80% power required to detect Kappa values significantly different from 0.40 was 85 impacted canines [25]. We selected a total of 105 canines to anticipate any possible measuring complication.

All the measurements were statistically analyzed using SPSS Statistics version 19 (SPSS Inc., Chicago, IL) software.

## 3. Results

### 3.1. Inter- and Intrarater Agreement

Cohen's Kappa values, obtained comparing *t*_0_ and *t*_1_, were the following: between 0.803 and 0.956 for KPG index, indicating an almost perfect intrarater agreement; between 0.786 and 0.922 for Ericson and Kurol's analysis, indicating substantial or in some cases almost perfect intrarater agreement; between 0.691 and 0.879 for Stewart's measurement, indicating substantial or in some cases almost perfect intrarater agreement.

Kendall's* W* values were the following: 0.967 at *t*_0_ and 0.989 at *t*_1_ for the KPG index, thus demonstrating an almost perfect interrater statistical agreement; 0.801 at *t*_0_ and 0.892 at *t*_1_ for Ericson and Kurol's analysis, thus demonstrating an almost perfect interrater statistical agreement; 0.775 at *t*_0_ and 0.844 at *t*_1_ for Stewart's measurement, thus demonstrating a substantial or in some cases almost perfect interrater statistical agreement.

### 3.2. 2D and 3D Indexes Agreement

Table 1 shows the comparative results regarding the prediction of treatment duration with KPG index and Stewart's measurement of canine's cusp tip vertical distance from occlusal plane. Considering Stewart's measurement as the reference standard, the sensitivity of KPG index was 0.846, while the specificity and negative predictive values were both 0.556. There was a statistically significant (*P* < 0.05) moderate (*r* = 0.402) association between the results obtained with both analyses, but conversely it was not possible to reject their independence at a strong statistically significant level (*P* = 0.053).

Table 2 shows the comparative results regarding the prediction of treatment difficulty degree with KPG index and Ericson and Kurol's analysis of canine's cusp tip position relative to the lateral incisor bisecting axis. Considering Ericson and Kurol's analysis as the reference standard, the sensitivity of KPG index was 0.941, while the specificity and negative predictive values were 0.444 and 0.889, respectively. There was a statistically significant (*P* < 0.01) moderate (*r* = 0.441) association between the results obtained with both analyses and a rejection of independency at a statistically significant level (*P* < 0.05).

Table 3 shows the comparative results between Stewart's measurement and Ericson and Kurol's analysis. Considering Ericson and Kurol's analysis as the reference standard, the sensitivity of Stewart's measurement was 0.824, while the specificity and negative predictive values were 0.333 and 0.667, respectively. There was no statistically significant (*P* = 0.303) association between the results obtained with both analyses, and it was not possible to reject their independence at a statistically significant level (*P* = 0.500).

## 4. Discussion

Orthodontic treatment of impacted canines is an interesting and absorbing challenge for every orthodontist, both from the diagnostic and the therapeutic point of view [26]. Several techniques were suggested to prevent, intercept or actively treat impacted maxillary canines, depending on patient age, canine position, presence of a malocclusion, and conditions of surrounding teeth [27–30].

Sometimes the final therapeutic decision (canine extraction or orthodontic traction; type and timing of orthodontic traction) could be a quandary for both the patient and the orthodontist, and in these cases treatment duration and difficulty degree are factors of crucial importance to considerate: for this reason, several authors tried to elaborate different methods to estimate them, utilizing radiographic images such as OPG, occlusal, periapical, and lateral cephalograms [31, 32].

OPG evaluation is the most common clinical approach used by orthodontists as first screening radiological exam, which is why we decided to focus our interest on OPG derived indexes. We tested the agreement of KPG index with these well-known 2D indexes as a first step in its validation process.

Unfortunately, several factors could affect 2D images quality and accuracy, due to patient positioning errors or even to distortion effects inherent to the radiological technique used. In order to limit these confounding factors, aiming to evaluate the efficacy of a prognostic index, in several studies only one radiologist was allowed to perform all radiological exams, always with the same equipment. We decided to test the effectiveness of these indexes; therefore, we included radiological images coming from different radiological centers, utilizing different equipment: this allowed us to simulate everyday conditions occurring in an orthodontic practice, where radiological images origin could be rather heterogeneous and could also explain the difference that we found in our study regarding intra- and interrater reliability of 2D indexes, even if also some other studies pointed out this possible lack of accuracy when using 2D radiological images. On the other hand, high quality protocols adopted by the radiological centers involved in the present study, thus producing radiological images with a very low incidence of technical errors, helped us to limit this confounding effect when assessing these indexes performance.

Nevertheless, as reported by several authors, the reliability of OPG in the anterior maxilla is limited: an overestimation of impacted canines angle and distance compared to the midline is generally present; furthermore, in patients with small interincisors angles or with an important intermaxillary discrepancy, apical or coronal parts of anterior teeth could appear out of focus or even invisible [33]. Finally, images alteration along the horizontal plane tends to be nonlinear [34] and also vertical measurements are not completely reliable [35].

This could explain why some measures were found to be related to treatment duration or difficulty degree only in some studies, while they were considered noninfluential by some others: if canine position has an important role in determining treatment peculiarity, it must be determined without imaging errors that act as confounding factors [36, 37]. For this reason, a 3D radiographic technique such as CBCT, thanks to the accuracy of its derived measurements, is of critical importance in exactly determining impacted canines position, and an index based on these images could be more reliable compared to those based on 2D dataset.

Stewart found that the greater the distance that the canine must move to correctly erupt, the longer the treatment will take; he was aware that the third dimension of the anterior maxilla cannot be seen on an OPT, and then he hypothesized that the more vertically displaced the impacted canine is, the longer could be this distance. Finally he concluded that 3D radiological techniques use could allow us to better understand how the position of an impacted canines relates to treatment duration.

In our study, we found a weaker correlation between Stewart's measurement and KPG index, compared to Ericson and Kurol's analysis. This could be due to the fact that the vertical position of canine's cusp tip is only one of the six factors considered by the KPG index: consequently its contribution to the overall index could be masked by the remaining five. Furthermore, the threshold of 14 mm between shorter and longer treatments was found only after data analysis, and it was not hypothesized during the study design, based on clinical or theoretical evaluations: not being hypothesis driven, the results of this study could be biased by accidental characteristics of the analyzed sample.

Otherwise, Ericson and Kurol's analysis was based on a prospective clinical trial; after that they found that spontaneous eruption of impacted canines with the crown tip mesial to the lateral midline was significantly less likely to happen after corresponding primary canine extraction, compared to more distal ones. Moreover, due to anatomical factors, canine angulation tends to increase while it migrates more mesially: this fact has an impact on root apex *x* and *y* scores when rating KPG index; then it could explain why the concordance between these two indexes is higher.

We also fund that Ericson and Kurol's analysis results and Stewart's measurements were not significantly associated: this could seem obvious, considering that the first one was conceived to evaluate treatment difficulty, whereas the second one aimed to predict treatment duration. Nevertheless, it must be considered that usually more complex impacted canines need longer treatments in order to be driven in their correct position.

Finally, CBCT images are of fundamental importance in recognizing the presence of adjacent teeth root resorption, impacted canines root anomalies, and possible overlap between canine's crown and incisor's roots, even if there is not yet an agreement regarding their usefulness in planning canine's surgical exposure and direction of active orthodontic traction [38–41].

Undoubtedly, the retrospective design of most of the studies that tried to correlate canine position with treatment duration and difficulty degree contributed to weaken 2D indexes reliability. Several factors, which in a retrospective study are difficult to control, could influence treatment development: age, malocclusion complexity degree, number of failed appointments and orthodontic appliances breakages, oral hygiene maintenance, patient compliance, and treatment protocol.

An appropriately designed prospective clinical trial, taking into account and monitoring all of these confounding factors, will be able to find a stronger evidence regarding factors influencing impacted maxillary canines treatment duration and difficulty level, allowing us also to clinically validate the KPG index or, if it is not the case, to correct it or to elaborate a new reliable 3D index, accounting for canine's real spatial position influence on them.

## 5. Conclusions

Our results demonstrate the following:

- Ericson and Kurol's analysis and Stewart's 2D indexes for predicting impacted maxillary canines treatment duration and difficulty sometimes are discordant;
- intra- and interrater agreement are higher for KPG index, when compared to these 2D indexes;
- the KPG index, considering the canine position in all the three dimensions, allows us to exactly evaluate the distance of the crown from the ideal position.

## Figures and Tables

**(a):** 

KPG	Stewart		Total
	Shorter	Longer	
Shorter	66	12	78
Longer	12	15	27

Total	78	27	105

**Table:** (b) Chi-square tests

	Value	*P* value
Pearson correlation (*r*)	0.402	0.017∗
Yates *χ* ^2^	3.741	0.053
Positive likelihood ratio	1.904	
Negative likelihood ratio	0.277	
Sensitivity	0.846	
Specificity	0.556	
Positive predictive value	0.846	
Negative predictive value	0.556	

∗Statistically significant association.

**(a):** 

KPG	Ericson and Kurol		Total
	Easy	Difficult	
Easy	48	30	78
Difficult	3	24	27

Total	51	54	105

**Table:** (b) Chi-square tests

	Value	*P* value
Pearson correlation (*r*)	0.441	0.008∗
Yates *χ* ^2^	4.937	0.026^†^
Positive likelihood ratio	1.694	
Negative likelihood ratio	0.132	
Sensitivity	0.941	
Specificity	0.444	
Positive predictive value	0.615	
Negative predictive value	0.889	

∗Statistically significant association.

^†^Statistically significant rejection of independency.

**(a):** 

Stewart	Ericson and Kurol		Total
	Easy	Difficult	
Shorter	42	36	78
Longer	9	18	27

Total	51	54	105

**Table:** (b) Chi-square tests

	Value	*P* value
Pearson correlation (*r*)	0.179	0.303
*χ* ^ 2^	0.455	0.500
Positive likelihood ratio	1.235	
Negative likelihood ratio	0.529	
Sensitivity	0.824	
Specificity	0.333	
Positive predictive value	0.538	
Negative predictive value	0.667	
